# The decay of HIV under anti-retroviral therapy is biphasic even in humanized mice with just T cells

**DOI:** 10.1128/jvi.01321-25

**Published:** 2025-12-30

**Authors:** Jasmine A. F. Kreig, Angela Wahl, Elisabete Fernandes, Jenna B. Honeycutt, J. Victor Garcia, Ruy M. Ribeiro

**Affiliations:** 1 Theoretical Biology and Biophysics, Theoretical Division, Los Alamos National Laboratory 5112, Los Alamos, New Mexico, USA; 2 Department of Microbiology, University of Alabama at Birmingham 318277, Birmingham, Alabama, USA; 3 Faculdade de Medicina, Universidade de Lisboa 37809, Lisbon, Lisbon, Portugal; 4 Division of Infectious Diseases, Center for AIDS Research, The University of North Carolina at Chapel Hill 219040 , Chapel Hill, North Carolina, USA; University Hospital Tübingen, Tübingen, Germany

**Keywords:** humanized mice, HIV-1 decay, cellular tropism, biphasic decline

## Abstract

HIV-1 plasma viral load decays in a biphasic manner during antiretroviral therapy (ART). It was hypothesized that this is due to infection of different cell types, namely CD4+ T cells and macrophages. We studied this possibility directly by modeling the decay of HIV-1 in humanized mice. We utilized previously published data from humanized T-cell only mice (TOM) and myeloid-only mice (MOM) infected with HIV-1 and treated with a potent ART regimen. Viral load decay dynamics were modeled using either a single or a biexponential decay fitted using nonlinear mixed effects techniques. Fits were compared using the corrected Bayesian information criterion (BICc). In TOM, the biphasic model was significantly better than a single-phase decay model (ΔBICc ≈ 16) despite additional parameters. In MOM, the biphasic decay was statistically better, but there was substantial uncertainty because the virus goes below detection very fast. The first-phase half-life was consistent between groups (1.2 days in MOM and 1.3 days in TOM) and similar to the half-life estimated in human infection. The second-phase decay in these mice was minimal likely due to low initial viral loads. Additional analyses with mice containing both CD4+ T cells and macrophages or X4-tropic virus-infected MOM mice confirmed the biphasic pattern, demonstrating the robustness of this result. The biphasic decline in HIV-1 occurs, even with only CD4+ T cells, refuting the hypothesis that distinct cell populations (CD4+ T cells and macrophages) drive each decay phase. These findings support an alternative model in which the observed dynamics arise from intrinsic properties of the viral infection lifecycle rather than from cellular compartmentalization.

**IMPORTANCE:**

It is well known that when antiretroviral therapy is started in people infected with HIV, the decay of virus in the periphery is biphasic early on (followed by other slower phases). One possibility for this pattern of decay is infection of two different types of cells (suggested previously to be CD4+ T cells and macrophages), with different turnovers giving rise to the biphasic decline. We addressed this issue directly in a humanized mouse model of HIV, taking advantage of mice reconstituted with just T cells and treated with antiretroviral drugs. We found that the observed decay is biphasic, which eliminates the hypothesis that the biphasic decline is due to the co-existence of the two types of cells. It is possible that integration dynamics, as we previously proposed, are responsible for the observed biphasic decline.

## INTRODUCTION

HIV infects cells by engaging the glycoprotein CD4 (cluster of differentiation 4) on the surface of cells and a co-receptor, often C-C chemokine receptor type 5 (CCR5) or C-X-C chemokine receptor type 4 (CXCR4) (1). The main cellular targets of HIV in the body are CD4+ T cells and macrophages, where these proteins are co-expressed (1). A powerful tool in the study of infection of these different subsets of cells is humanized mouse models, that is, chimeras consisting of immunodeficient mice engrafted with human hematopoietic stem cells (2, 3). There are different humanized mouse models, including mice containing both T-cells and myeloid cells, mice with just T-cells and no myeloid cells, and vice versa (see a recent review in [4]). All these models support productive HIV infection and were used to study multiple aspects of infection, including the contribution of macrophages (5), central nervous system infection (6, 7), HIV persistence (8–10), HIV latency and reversal (11, 12), pharmacological interventions (13), and others (14, 15).

In humans, treatment of HIV infection with potent antiretrovirals results in a biphasic decline of the virus in the periphery, with a fast first phase half-life (time needed to remove half of the virions in circulation) of about 1 day and a slower second phase half-life of ~21 days (16–18). These dynamics have been consistently observed with many classes of antiretrovirals (19). One mechanism that has been evoked to explain the existence of two phases of decay is viral infection in different cell compartments, with different decay rates. One possibility is that the first phase is driven by the decay of infected CD4+ T cells and the second phase by the loss of infected macrophages (20, 21). This hypothesis can be directly tested in the appropriate humanized mouse models of HIV infection.

Here, we use data from humanized mice treated with antiretrovirals to analyze in detail the decay of HIV-1 under treatment. We take advantage of mice possessing only T cells (T-cell only mouse, TOM) or only myeloid cells, such as macrophages (myeloid-only mouse, MOM), to investigate whether the decay of virus is biphasic or exhibits a single phase, as would be predicted by the hypothesis that the biphasic decay is due to different types of cells, specifically T cells and macrophages.

## MATERIALS AND METHODS

### Data

The data used in this study were published previously (5, 8, 11). Briefly, humanized T-cell only mice (TOM) were generated by implanting human thymus and liver tissue under the kidney capsule of sublethally irradiated NOD.Cg-*Prkdc^scid^ Il2rgtm^1Wjl^*/SzJ mice (NSG; The Jackson Laboratory). Bone marrow/liver/thymus (BLT) mice also received autologous CD34+ hematopoietic stem cells (CD34 Microbead Kit, catalog 130-046-703; Miltenyi Biotec). Humanized MOMs were created by transplanting sublethally irradiated NOD.CB17-*Prkdc^scid^*/J mice (NOD/SCID; The Jackson Laboratory) with approximately 1 × 10^6^ cord blood or liver-derived CD34+ hematopoietic stem cells. Previous flow cytometric analysis confirmed the absence of human myeloid cells in ToM and human T cells in MoM (5, 8, 22). Moreover, MoM are constructed in a NOD/SCID background, and the thymus of NOD/SCID mice is unable to support thymopoiesis.

Mice were infected with R5-tropic or X4-tropic HIV viruses at a dose of 360,000 tissue culture infectious units (TCIU), as described before (5, 8). To measure viral load during treatment, peripheral blood was collected serially, and plasma was isolated by centrifugation. HIV infection was monitored in peripheral blood plasma with a one-step reverse transcriptase real-time PCR assay (ABI custom TaqMan Assays-by-Design, with an assay sensitivity of 668 RNA copies/mL) (5). Mice were administered daily intraperitoneal injections of emtricitabine (FTC; 211 mg per kg body weight), tenofovir disoproxil fumarate (TDF; 205 mg per kg body weight), and raltegravir (RAL; 56 mg per kg body weight), two reverse transcriptase inhibitors, and an integrase inhibitor.

Animal protocols were approved by the Institutional Animal Care and Use Committee of the University of North Carolina at Chapel Hill.

### Model

A standard and well-tested model of HIV-1 infection and treatment (20) is to consider two types of target cells, *T* and *M*, which have been associated with CD4+ T cells and macrophages (20, 23–25). These cells are infected at constant rates (*k* and *k_M_*) to generate two types of infected cells, short-lived (T*) and longer-lived (M*), which then produce virus at different rates (*P* and *p_M_*). The virus is cleared at a constant rate, *c*, which has been measured to be very fast (*c*>20/day) (26). Treatment is modeled as a reduction in infection rate by (1−ε), where ε is the effectiveness of the drug. This model can be written as:


$$\begin{array}{rl} & \frac{dT^{*}}{dt}=\left(1-\epsilon_{T}\right)kVT_{0}-\delta T^{*}\\ & \frac{dM^{*}}{dt}=\left(1-\epsilon_{M}\right)k_{M}VM_{0}-\mu_{M}M^{*}\\ & \frac{dV}{dt}=pT^{*}+p_{M}M^{*}-cV \end{array}$$

where we have assumed, as often done (20), that the number of target cells remains approximately at a constant level (*T_0_* and *M_0_*) during the period of short-term therapy that we analyze here. If, in addition, we assume that ART is very potent and the number of new infections during therapy is negligible (ε_M_ = ε_T_ = 1), then those equations can be solved to show that the viral load changes according to


$$V\left(t\right)=V_{0}\left(Ae^{-\delta t}+Be^{-\mu_{M}t}+\left(1-A-B\right)e^{-ct}\right)$$

with $A=\frac{pkT_{0}}{\delta \left(c-\delta \right)}$ and $B=\frac{c\delta -pkT_{0}}{\delta \left(c-\mu_{M}\right)}$. Since *c* is large (e.g., *c* > 20/day^26^), the last term (~*e ^ct^*) becomes very small quickly. Indeed, our data have a weekly frequency, and at 1 week, this term corresponds to <10^−30^. Thus, the solution for *V(t*) becomes a simple biexponential, with two slopes of decay corresponding to the turnover of the two types of infected cells (T* at δ and M* at µ_M_). Moreover, at time *t* = 0, *V*(0) = *V*_0_, which implies that *A + B ≈ 1*. This simplified model is what we fit to our data set of humanized mice infected with HIV and treated with the potent ART regimen. That is, $V(t)=V_{0}(Ae^{-\delta t}+\left(1-A\right)e^{-\mu_{M}t})$.

This model has three parameters – A, δ, and *µ*_M_ – and allows us to test whether the decline is single (A=1) or biphasic (A≠1) and to estimate the resultant decay rates. From these, we calculated the first- and second-phase half-lives as log(2)/δ and log(2)/µ_M_, respectively.

### Data fitting

We fitted the model using a population approach (non-linear mixed effects) in Monolix (Lixoft SA, Antony, France) to a data set of infected MOMs (*n* = 78) and TOMs (*n* = 8). In multiple cases, viral load quickly goes below detection in the treated mice, and we do not include datapoints below the level of detection (LoD=688 RNA copies/mL) after the first instance of such a datapoint, which is used as censored in the fitting algorithm.

Assuming a constant error model, we fit the log_10_ of the viral load data for MOMs and TOMs separately because of the imbalance in group size. We use the corrected Bayesian information criteria (BICc) to evaluate each fit, wherein the lower the BICc value, the better the fit (27). We also assessed what is the most parsimonious structure for the random effects of the parameters using BICc. We present only the best resulting model. As a further test of the robustness of our main result, we also fitted mice that have both T-cells and myeloid cells (BLT mice) and MOM mice infected with an X4-tropic virus.

## RESULTS AND DISCUSSION

When HIV-1 infection is treated with potent antiretroviral therapy, an initial biphasic decline is observed. We tested whether this observation holds true in humanized mice reconstituted only with T cells (TOM) or only with myeloid cells (MOM), which without treatment can maintain infection with high viral loads for more than 10 weeks (5, 11). If the hypothesis that the two phases of decline at the start of treatment are due to the decay of infected CD4+ T cells and macrophages at different rates, one would expect that in each of these mice types, a single phase of decline at different rates would be observed.

**Fig 1:**
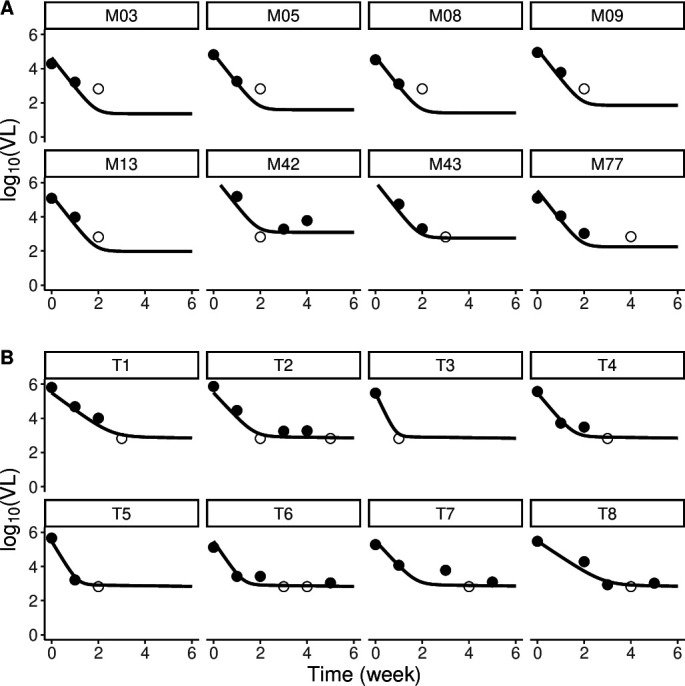
Bi-phasic fits for (**A**) selected MOM and (**B**) all TOM mice. Each panel represents one mouse’s viral load data (symbols) and corresponding best fit (line). Data below the limit of detection are represented as open circles. (We only present a subset of the MOM-infected mice.)

We fitted the observed decay in viral load in these mice upon initiation of treatment using a simpler version of standard models of viral infection (20) (see Methods). Our fits to the data showed that the biphasic model performed better statistically than the single decay model (Table 1; Fig. 1) across both groups of mice (TOM and MOM), with improvements of BICc of ~19 units in MOM and ~16 units in TOM despite the extra parameters for the biphasic model. However, in MOM, this biphasic decline is not visually apparent in the limited data, and it could be driven by the censored data. Only MOM number 42 shows the slowing down of decline characteristic of biphasic decay.

**TABLE 1:** Best single and biphasic fits to viral load data in humanized mice^*a*^^,^^*b*^

		MOM (*N* = 78)	TOM (*N* = 8)	BLT (*N* = 33)	X4 (*N* = 19)
Single	BICc	331.3	90.1	310.4	120.6
	Random effects	$\delta ,V_{0}$	$V_{0}$	$\delta ,V_{0}$	$\delta ,V_{0}$
	A	1	1	1	1
	δ (/week)	3.9 (3.6, 4.2)	3.5 (2.4, 5.1)	1.9 (1.6, 2.2)	2.1 (1.5, 4.0)
	First-phase half-life (days)	1.2 (1.1, 1.4)	1.4 (0.96, 2.0)	2.6 (2.2, 3.0)	2.3 (1.6, 3.3)
	$\mu_{M}$	NA	NA	NA	NA
	$V_{0}$ (log_10_)	4.7 (4.6, 4.9)	4.9 (4.2, 5.6)	5.5 (5.3, 5.7)	4.4 (4.0, 4.8)
Biphasic	BICc	312.8	74.0	264.4	111.7
	Random effects	$V_{0}$	δ	$V_{0}$	$\mu_{M},V_{0}$
	A	0.999 (0.999, 1)	0.998 (0.996, 1)	0.997 (0.994, 0.999)	0.999 (0.998, 1)
	δ (/week)	4.0 (3.7, 4.3)	3.7 (2.4, 5.8)	2.6 (2.3, 2.9)	3.6 (3.1, 4.1)
	First-phase half-life (days)	1.2 (1.1, 1.3)	1.3 (0.84, 2.0)	1.9 (1.7, 2.1)	1.4 (1.2, 1.6)
	$\mu_{M}$ (/week)	0.000224 (0, 3.28e+43)	6.37e−05 (0, 8.78e+24)	0.284 (0.151, 0.536)	3.83e−5 (5.24e−14, 2.8e+4)
	Second-phase half-life (weeks)	3094	10,881	2.4	18,098
	$V_{0}$	4.8 (4.6, 4.9)	5.5 (5.2, 5.8)	5.7 (5.5, 5.9)	4.8 (4.5, 5.2)

^*a*^ Each column presents the results of fitting for myeloid-only mice (MOM), T cells-only mice (TOM), bone marrow/liver/thymus mice (BLT) all these infected with R5-tropic HIV virus, and MOM mice infected with X4-tropic virus (X4). The top of the table shows results for single decay fits and the bottom for the biphasic fits. Each row shows the BICc value, the parameters with random effects, parameter values with 95% confidence intervals, and half-life of the first and second phases of decline (when applicable). In all cases, the data support models with a biphasic decline. First-phase half-life is calculated as log(2)/δ and second-phase half-life as log(2)/µ_M_.

^*b*^ “NA” indicates not applicable.

Importantly, we estimated that the first phase of decay has a similar half-life of 1.2 days for MOM and 1.3 days for TOM, and these are relatively well-constrained with 95% confidence intervals (1.1, 1.3) days and (0.84, 2.0), respectively. Moreover, the single-phase decay model estimates half-lives similar to the ones shown above (Table 1). On the contrary, the second phase of decline is much slower, with best estimates indicating essentially no decay in both MOM and TOM. It is possible that this result is due to difficulties estimating the second phase of decay because the virus is very low in this phase. This would be a bigger issue for MOM that starts with a lower viral load (V_0_= 4.8) than TOM (V_0_= 5.5) (*P* < 2.2e−16).

To understand our results better, we also fitted BLT mice, which have both CD4 T cells and macrophages and MOM mice infected with X4 virus. Similar to both MOM and TOM, the biphasic model performed better statistically than the single model (Table 1) demonstrating BICc improvements of ~46 and ~9 units in BLT and MOM-X4, respectively. But again, in MOM-X4, this could be driven by censored data, and only one mouse showed a clear slowing down of decay (not shown).

The fits presented in Table 1 represent the scenarios that yielded the lowest BICc values for each type of model. For comparison, we include information on alternative random effects structures (Table 2) across all mouse groups and for both the single and biphasic models. This table shows that, even if we define the same random effects for single phase and biphasic model (e.g., on δ and *V*_0_), the biphasic model has better statistical support.

**TABLE 2:** BICc values for alternative scenarios with different random effects structures for all mice groups and both single and biphasic models

	Random effects	MOM (*N* = 78)	TOM (*N* = 8)	BLT (*N* = 33)	X4 (*N* = 19)
Single decay model	δ	350.51	91.28	318.12	120.74
	$V_{0}$	365.94	90.1	356.83	127.23
	$\delta ,V_{0}$	331.35	93.12	310.44	120.64
Biphasic model	A,δ	360.27	80.6	315.6	133.41
	$A,V_{0}$	355.01	80.48	267.64	120.25
	$\delta ,V_{0}$	317.1	80.61	305.22	127.52
	$A,\delta ,V_{0}$	327.52	82.74	311.69	116.97

Taken together, these results indicate that a model with biphasic decline for the viral load upon treatment initiation describes the data better in TOM and possibly in MOM as well. In addition, we find that the first phase decay rate and corresponding half-lives are very similar in TOM and MOM, independent of whether the latter have a true biphasic or only a single-phase decay. This suggests that the original hypothesis that the two phases of decline correspond to infection of these two different types of cells that are lost at different rates is untenable. Note that the estimated second phase decay rate in BLT mice (t _1/2_= 2.4 weeks) is also much slower than the first phase decay rate of macrophages in MOM (Table 1).

We have proposed an alternative viral dynamical model, which provides a better description of the dynamics of HIV-1 under integrase strand-transfer inhibitor-containing ART (28), where the first and second phase are due to the same type of cells with different integration half-lives possibly due to different states of cell activation on infection (29, 30). This model could explain the present data where mice with just T-cells, nevertheless, show biphasic decay of viral load with a treatment that includes an integrase inhibitor. The first phase seen in the present study with weekly sampling would correspond to phase 1b in that study in humans, with a half-life of 1.8 days, which is consistent with the 1.2–1.3 days found here in humanized mice. Phase 2 here corresponds to phase 2 of that study although the rates are different since in the previous study, the half-life of phase 2 was 5 weeks (28), and here, it is much longer. For MOM, the same phenomenon could be operative in macrophages leading to a biphasic decline, or alternatively, there is much less variability in integration rates in macrophages, which would explain a single phase of decline. It should be pointed out that others have also cast doubt on turnover of infected macrophages being the source of the second phase decline by studying timings of the HIV intracellular lifecycle (24). This explanation based on different integration rates has support from human infection and treatment (28), but variability in other phases of the replication cycle may also explain the observed biphasic decline.

Although humanized mice have been used extensively for HIV research (4), their infection is not the same as human infection; thus, it is possible that different mechanisms are operating in humans. However, the reconstitution of MOM and TOM with human cells and the similar kinetics of virus before and under treatment is an indication that these mice are a good representation of similar processes in human infection. Our study has other limitations, perhaps the most important of which is that the initial viral load in these mice is relatively low, making it difficult to estimate the rate of the second phase of decay with confidence, and indeed, we found large confidence intervals for this estimate. This also makes asserting biphasic decline in MOM difficult. Nevertheless, the simpler results of biphasic decline in TOM and similar first phase half-lives in both MOM and TOM are robust. Another limitation is that we have a small number of TOM mice in our study. Although it would be better to have a more balanced number of mice, and that is the reason why we fitted the two groups separately, the results demonstrate that we have power to detect the statistical improvement of using a biphasic model in TOM (as seen in the BICc values). Finally, one assumption that we made is that the clearance rate of free virus, *c*, is large in the infected mice, as has been estimated in infected people (26). If it was much slower, than it would be possible that the first phase of decay observed in both MOM and TOM mice (t_1/2_ ~1.2–1.3 days) is due to viral clearance, and the second phase would correspond to the turnover of infected cells. However, this seems very unlikely for several reasons: (i) this hypothesis would imply that the turnover of productively infected cells, which would correspond to the second phase, is much slower than measured before, even though these are human cells infected by HIV; (ii) it would also imply that the similarity of the first phase decay rate with that observed in humans is only a (remarkable) coincidence; and iii) virus clearance in mice is typically fast, as seen for other viruses (31–33).

Overall, modeling the decay of HIV viral load in infected humanized mice under potent antiretroviral therapy indicates that the observed biphasic decline in humans is not due to different populations (lineages) of infected hematopoietic cells, but rather it is an intrinsic dynamical process of infection of CD4+ T cells and, possibly, macrophages.

## Data Availability

The data in this study will be made available by the authors to interested scientists upon reasonable request.
